# An exploratory simulation study and prediction model on human brain behavior and activity using an integration of deep neural network and biosensor Rabi antenna

**DOI:** 10.1016/j.heliyon.2023.e15749

**Published:** 2023-04-25

**Authors:** Nhat Truong Pham, Montree Bunruangses, Phichai Youplao, Anita Garhwal, Kanad Ray, Arup Roy, Sarawoot Boonkirdram, Preecha Yupapin, Muhammad Arif Jalil, Jalil Ali, Shamim Kaiser, Mufti Mahmud, Saurav Mallik, Zhongming Zhao

**Affiliations:** a Computational Biology and Bioinformatics Laboratory, Department of Integrative Biotechnology, College of Biotechnology and Bioengineering, Sungkyunkwan University, Suwon 16419, Gyeonggi-do, Republic of Korea; b Department of Computer Engineering, Faculty of Industrial Education, Rajamangala University of Technology Phra Nakhon, Bangkok 10300, Thailand; c Department of Electrical Engineering, Faculty of Industry and Technology, Rajamangala University of Technology Isan Sakon Nakhon Campus, 199 Village no. 3, Phungkon, Sakon Nakhon 47160, Thailand; d Asia Metropolitan University, 6, Jalan Lembah, Bandar Baru Seri Alam 81750, Masai, Johor, Malaysia; e Amity School of Applied Sciences, Amity University Rajasthan, Jaipur, India; f Facultad de CienciasFisico-Matematicas, Benemérita Universidad Autónoma de Puebla, Av. San Claudio y AV. 18 sur, Col. San Manuel Ciudad Universitaria, Pueble Pue. 72570, Mexico; g Faubert Lab, Ecole d'optométrie, Université de Montréal, Montréal, QC H3T1P1, Canada; h School of Computing and Information Technology, Reva University, Bengaluru, Karnataka 560064, India; i Program of Electrical and Electronics, Faculty of Industrial Technology, Sakon Nakhon Rajabhat University, 680 Nittayo, Mueang, Sakon Nakhon 47000, Thailand; j Department of Electrical Technology, School of Industrial Technology, Sakonnakhon Technical College, Institute of Vocational Education Northeastern 2, Sakonnakhon 47000, Thailand; k Department of Physics, Faculty of Science, Unversiti Teknologi Malaysia, 81310 Skudai, Johor, Malaysia; l Institute of Information Technology, Jahangirnagar University, Savar, Dhaka 1342, Bangladesh; m Nottingham Trent University, Clifton Lane, NG11 8NS, Nottingham, United Kingdom; n Department of Environmental Health, Harvard T H Chan School of Public Health, Boston, MA 02115, USA; o Center for Precision Health, School of Biomedical Informatics, The University of Texas Health Science Center at Houston, Houston, TX 77030, USA; p Human Genetics Center, School of Public Health, The University of Texas Health Science Center at Houston, Houston, TX 77030, USA

**Keywords:** Brain neural network, Deep brain sensors, Brain-Rabi antenna, Deep learning, Biosensors on human brain/action, Simulation, Sensitivity

## Abstract

The plasmonic antenna probe is constructed using a silver rod embedded in a modified Mach-Zehnder interferometer (MZI) ad-drop filter. Rabi antennas are formed when space-time control reaches two levels of system oscillation and can be used as human brain sensor probes. Photonic neural networks are designed using brain-Rabi antenna communication, and transmissions are connected via neurons. Communication signals are carried by electron spin (up and down) and adjustable Rabi frequency. Hidden variables and deep brain signals can be obtained by external detection. A Rabi antenna has been developed by simulation using computer simulation technology (CST) software. Additionally, a communication device has been developed that uses the Optiwave program with Finite-Difference Time-Domain (OptiFDTD). The output signal is plotted using the MATLAB program with the parameters of the OptiFDTD simulation results. The proposed antenna oscillates in the frequency range of 192 THz to 202 THz with a maximum gain of 22.4 dBi. The sensitivity of the sensor is calculated along with the result of electron spin and applied to form a human brain connection. Moreover, intelligent machine learning algorithms are proposed to identify high-quality transmissions and predict the behavior of transmissions in the near future. During the process, a root mean square error (RMSE) of 2.3332(±0.2338) was obtained. Finally, it can be said that our proposed model can efficiently predict human mind, thoughts, behavior as well as action/reaction, which can be greatly helpful in the diagnosis of various neuro-degenerative/psychological diseases (such as Alzheimer's, dementia, etc.) and for security purposes.

## 1 Introduction

Nowadays, the diagnosis of various neuro-degenerative and psychological diseases through multi-disciplinary approaches is one of the topics of current research interest [1], [2]. Multi-disciplinary approaches include machine learning, neural networks or deep learning, biosensor, bioelectronics, and many more. Our main goal is to focus on human mind, thoughts, behavior, and action/reaction. Therefore, our current work is essentially an extensive simulation study and a corresponding disease prediction model on the human brain through the integration of deep neural network and biosensor Rabi antennas, targeting on human mind, thoughts, behavior and actions.

In general, super-sensitive quantum sensors are created for brain tissue that might be able to detect any brain disorders or diseases such as Alzheimer's, Dementia, Parkinson's disease, and even brain cancer by identifying the spots across the entire brain where the respective traversing speed of the signals is significantly slowing down [3]. In soft computing, a neural network (or deep learning) is basically a tool that consists of four major components: inputs, weight factors, a bias, and an output [4]. The data is passed from one layer to the next nearest neighboring layer, denoted as a feed-forward neural network. Moreover, a neural network can make complex decisions based on the output of previously taken decisions (or layers). Interestingly, a photonic neural network is designed using brain-Rabi antenna communication, while the corresponding transmission is connected by neurons.

The Rabi antenna is a device designed using a modified add-drop multiplexer that relies on low-level energy oscillations of a two-level system. In recent years, Rabi optical vibration has been studied as a coherent nonlinear interaction between light and matter. When a two-level atomic system with exciton resonances is introduced inside microgravity, the coupling between the two-level transitions due to resonance and cavity mode causes Rabi splits and Rabi oscillations. The electromagnetic field can be used to manipulate a single two-stage quantum arrangement by modulating its amplitude, frequency, and envelope curve. In this article, the Rabi antenna circuit is integrated with brain signal communication, which is transferred by neurons. The change in neuron energy affects the transmission signals within the network, and these signals can be measured and detected externally. Low-level neuron signals can be configured to represent human mind, thoughts, behavior and action/reaction signals, which can be distinguished by electron spins or Rabi frequency oscillations. The quantum neural network can be used to retrieve and recover hidden layer signals within the deep brain, which can be interpreted to generate human cognition and behavior.

**Figure 1:**
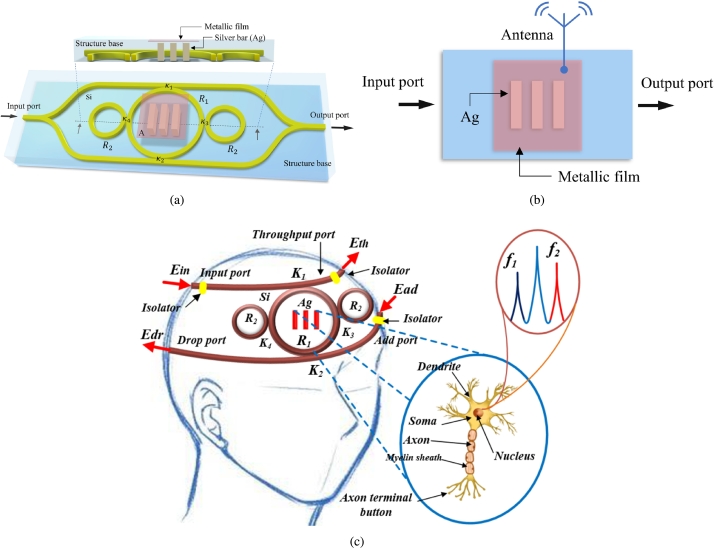
(a) Schematic diagram. Plasmonic antenna probe uses a silver bar embedded in a modified add-drop filter within a Mach Zehnder interferometer (MZI). (b) Equivalent circuit. (c) Rabi antenna probes for human mind, thoughts, behavior and action/reaction interpretation.

Electroencephalography (EEG) measures the electrical activity in the brain in the form of the flow of current in the neurons. It is a direct measurement method in neuroimaging. The measurement is carried out by placing the electrodes on the scalp. The measured data is then processed using amplifiers and analog/digital converters [5]. EEG signals are in the range of around 4 Hz to 100 Hz. Microring resonators have applications in various fields such as sensors, quantum communication, teleportation, and black hole [6], [7]. In addition, micro-electromechanical systems (MEMS) technology has shown great potential for various kinds of sensors [8], [9]. Rabi oscillation generation or Rabi antenna using a microring resonator is given by [10], [11]. Where the Rabi oscillation was generated without space-time. The present work consists of a space-time signal at the input port. In brain sensing, the transmitted signals from neurons are detected by electrodes. The electrodes are placed in the form of patches over the scalp. Low-voltage signals in the form of spikes are captured and converted into electronic signals. A review on brain sensing technologies is done by Robinson et al., where various sensing technologies such as electrical sensing and optical brain computer interfaces are discussed in detail [12]. Wearable dry electrode EEG sensing was presented, where multimodal EEG over the patient's head is recorded and analyzed. The technique used provided better results, and while capturing data patient also felt comfortable [13]. No surgery is needed to process the EEG signal using a brain-computer interface. A brain-computer interface (BCI) system requires a sensor, decoder, and actuator, which has applications in paralysis [14]. Along with brain-computer interface (BCI), computer-brain interface (CBI) techniques are also being developed. Conscious brain-to-brain (B2B) communication is performed using EEG, where data in the form of 0 and 1 are transmitted and received. Experimentally, two human minds communicated directly [15]. Another method of teleportation is using the space-time control method [16]. The Rabi antenna can have applications in human-like stereo sensors [17]. The human brain usually consists of cells that can communicate via wireless and wired connections and can transmit via plasmonic antennas or liquid-core waveguides. The electro-optical transducer (WGM) can penetrate the brain and cells at THz and probe the binding effect between electrons and brain cells to reflect the system. When light combines with the Au/Ag lattice, it in principle produces plasmon waves, and the collision of plasmon waves (plasma) causes dipole oscillations. Changes in the oscillation frequency (wavelength) of the sensor array through the brain cells can be examined by recognizing the output and interpreting the word. The interaction between plasma waves and brain cells can be observed at the sub-level using polarization patterns or output gate spin sensors. Various schemes, such as electrical, optical, and magnetic spin detection, can be applied to the output connection. The applied external modulation can also be changed via the additional port. The proposed system has the potential to be a quantum-level sensor, especially for the study of human mind, thoughts, behavior and action/reaction. In this work, a plasmonic Rabi antenna is designed and simulated using OptiFDTD and CST (computer simulation technology) software. The system consists of an MZI, and the center ring is embedded with silver (Ag) gratings. WGM is achieved, and Rabi antenna formation takes place. The designed antenna has applications in deep brain sensors. Fig. 1a shows the proposed system design. The future work will focus on EEG (electroencephalography) signals that can be applied at the modulation port of a microring system, where low-frequency EEG signals will be achieved for human mind, thoughts, behavior and action/reaction using the machine learning method.

## 2 Theoretical background

### 2.1 Plasmonic model

**Figure 2:**
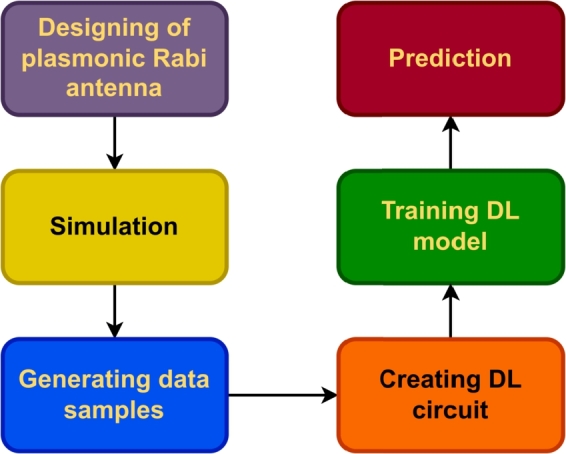
Flowchar of the process of the plasmonic Rabi antenna and DL circuits.

We propose using a plasmonic microring antenna network to sense changes in wavelength in the brain and a flat neural network (1 hidden layer) to process and interpret the output. More specifically, the proposed brain sensor circuit is realized by forming a flat distributed sensor network using distributed metamaterial antennas. Our motivation is to develop a brain-interface circuit device that overcomes limitations presented by bioelectrical signals, such as scalp electrode placement and data acquisition, EEG pattern variability, scalability to large populations, and ECG pattern heritability. Finally, we argue that the proposed system has potential for quantum-level sensors. Furthermore, applying deep learning circuits may even predict unknown signals and interpret dreams. Traditional approaches have not been able to achieve this. Based on the results of several experiments performed using novel deep learning (DL) circuits to predict dreams and develop unknown human-like brain sensors, signals can be encoded. Fig. 2 depicts a flowchart of the process of the plasmonic Rabi antenna and deep learning (DL) circuits. As shown in Fig. 2, the process of the plasmonic model is on the left side, while the process of the DL model is on the right side. First, the plasmonic Rabi antenna is designed and simulated to generate data samples. Then a DL circuit is constructed to train a DL model using the generated data. After that, the trained DL model is used for prediction.

The Rabi cycle in a two-level system is the variation of the population of transition states in a given optical field, which is proportional to the coupling intensity between the light and the atomic transition and the light field amplitude. Rabi oscillations between the two levels of a two-level system illuminate the resonant light where the transition occurs, at Rabi frequency. The incident light is tuned to the generalized Rabi frequency. The detuning of the incident light occurs at the generalized Rabi frequency. Fig. 1b shows the space-time distortion control circuit using silicon Mach Zehnder interferometer (MZI) embedded microring resonators. The silver bars are applied to the center ring resonator to form the antenna. The space-time light fields are input into the circuit at the MZI ports. The optical path difference of the traveling light fields within the two side rings can be controlled and vanished by the successive filtering operation. When the space-time distortion of light fields within the circuit has vanished, it results in the Rabi oscillation, where photons are squeezed from the circuit center. The embedded silver bars are coupled by the squeezed photons, where the dipole oscillation occurs. The plasma frequency is known as the Rabi frequency, which can be applied to the quantum antenna.

The input signal is the polarized laser [18], which is given by an equation (1).

(1) $$E_{in}=E_{0}\cdot exp(-ik_{z}z)$$

where $k_{z}=\frac{2\pi }{\lambda }$ is the wave number of the wave vector in the z-axis, *λ* is the wavelength, and $E_{0}$ is the initial amplitude of the field, and *z* is the propagation distance in the z-axis.

The propagation of light pulse within the nonlinear material and in refractive index (*n*) was given by [18] as shown in equation (2) below:

(2) $$n=n_{0}+n_{2}I=n_{0}+\frac{n_{2}}{A_{eff}}P$$

where $n_{0}$, $n_{2}$, *I*, *P*, and $A_{eff}$ are the linear and nonlinear refractive indices, optical intensity and optical power, effective core area, respectively.

The plasma WGM of electron oscillation is given using Drude model, which was given by [18] as shown in equation (3):

(3) $$\epsilon (\omega )=1-\frac{n_{e}e^{2}}{\epsilon_{0}m\omega^{2}}$$

where $n_{e}$, *e*, $\epsilon_{0}$ and *m* are electron density, electron charge, relative permittivity and mass of electron respectively. *ω* is angular frequency. Plasma frequency is given by equation (4):

(4) $$\omega_{p}=\sqrt{\frac{n_{e}e^{2}}{\epsilon_{0}m}}$$

The microring system was given by [18] as shown in equations (5) and (6):

(5) $$E_{th}=m_{3}E_{in}+m_{4}E_{add}$$

(6) $$E_{drop}=m_{5}E_{add}+m_{6}E_{in}$$

where $E_{th}$, $E_{in}$, $E_{add}$ and $E_{drop}$ are throughput port, input port, add and drop port respectively. $m_{3},m_{4},m_{5}$, and $m_{6}$ are constants, as given in [19].

The electron intensities at ports 1 and 2 are given by equations (7) and (8).

(7) $$\frac{I_{Th}}{I_{in}}={\left[\frac{E_{Th}}{E_{in}}\right]}^{2}$$

(8) $$\frac{I_{drop}}{I_{in}}={\left[\frac{E_{drop}}{E_{in}}\right]}^{2}$$

The Rabi frequency was given by [10] as shown in equation (9):

(9) $$F_{R}=|\frac{E_{0}\mu_{12}}{\frac{h}{2\pi }}|$$

Which is related to electric field equation of $(r)=E_{0}(z)=\vartheta A(z)e^{i\beta_{0}z}$, and $\vartheta ={\left(\frac{\mu_{0}}{\epsilon_{0}}\right)}^{\frac{1}{4}}(\sqrt{2n_{0}})$, where $\mu_{0}$ and $\epsilon_{0}$ permeability and permittivity in vacuum. $n_{0}$ is linear refractive index. $\beta_{0}=\frac{\omega n_{0}}{c}$ is the propagation constant, *c* is speed of light and A(z) is complex amplitude [10].

Following the MEMs concept, the circuit in Fig. 1c can be fabricated and formed as the sensing probe for deep brain sensors, in which the quantum antenna operation can be applied. The interference between the deep brain signals and antenna propagation fields can be probed and detected, where in this case the quantum signals are formed by spin wave propagation, which is obtained at the through and drop ports, respectively. The outputs are $90^{\circ }$ phase difference, which presents the two sides of time. The outputs in terms of spin density sensitivity are leveled by spin up (0) and spin down (1). In operation, we have proposed human mind, thoughts, behavior and action/reaction detection. Using the space-time distortion criteria, when the Rabi oscillation of the circuit in Fig. 1c occurred, the quantum antenna was in operation. Hence, deep brain signals are obtained. In application, we have proposed human mind, thoughts, behavior and action/reaction detection. When the Rabi oscillation occurs, the quantum antenna is in operation. The deep brain signals can be retrieved in terms of spin up and spin down, respectively. The use of a quantum sensor related to deep brain signals can be realized, which is useful for quantum deep learning and sensor applications.

### 2.2 Deep learning model

In this paper, we present a brain interface based on deep learning circuits using distributed metamaterial antennas. The idea is to create deep learning circuits from distributed metamaterial antennae and use spatiotemporal functional control to enable the interpretation of brain signals. The manuscript presents a distributed microantenna circuit configuration while reporting extensive evaluation results. The manuscript introduces the experimental setup, dataset, training, and evaluation processes. An optical neural network has been developed using brain-Rabi antenna communication. Transmission is connected through neurons. Communication signals are transmitted by electron spins (up and down) and adjustable Rabi frequencies. Hidden variables and deep brain signals can be obtained by external sensing. In particular, this work includes extensive simulation studies on the development of Rabi antennas. In addition, it provides intelligent machine learning algorithms to identify transmission quality and predict the behavior of transmission in the near future.

In this section, we aim to design a prediction model to forecast the time series or sequential data of the time domain of the Rabi Antenna. There are many methods that use forecasting time series data like simple exponential smoothing, smoothing average, auto-regressive integrated moving average, and others. With the development of neural networks and deep learning, recurrent neural network (RNN) [20] and its variants, especially long short-term memory (LSTM) [21], [22] network that has been investigated and put attention in many sequential problems, such as natural language processing, video processing, speech processing, time series, etc. On the other hand, although the RNN is also an artificial neural network for processing sequential data or time series, it suffers from the problem of vanishing and exploding gradients. Therefore, in this study, we focus on designing an LSTM network for time series prediction using time domain data of the Rabi antenna because it overcomes the limitation of RNN.

Long short-term memory is first presented by Hochreiter & Schmidhuber in [21] to overcome the vanishing and exploding gradients problem in RNN. Each LSTM cell can be computed and updated as equations (10)–(15):

(10) $$f_{t}=S_{g}(W_{f}X_{t}+U_{f}h_{t-1}+b_{f}),$$

(11) $$i_{t}=S_{g}(W_{i}X_{t}+U_{i}h_{t-1}+b_{i}),$$

(12) $$o_{t}=S_{g}(W_{o}X_{t}+U_{o}h_{t-1}+b_{o}),$$

(13) $${\overset{˜}{c}}_{t}=T_{g}(W_{c}X_{t}+U_{c}h_{t-1}+b_{c}),$$

(14) $$c_{t}=f_{t}⊙{\overset{˜}{c}}_{t-1}+i_{t}⊙{\overset{˜}{c}}_{t},$$

(15) $$h_{t}=o_{t}⊙T_{g}(c_{t}),$$

where $i_{t}$, $f_{t}$, $o_{t}$, $c_{t}$, $X_{t}$ and $h_{t}$ represent the input gate, forget gate, output gate, cell state with a self-recurrent, input vector, and hidden state at the time step *t*, respectively. Besides, $S_{g}$ and $T_{g}$ denote the sigmoid and tanh activation functions, the (⋅) operator is the element-wise product. The calculation inside the LSTM cell can be defined in four steps. Firstly, the forget state is computed as equation (10). Secondly, the input and output states are obtained in a similar way as equations (11) and (12), respectively. Thirdly, the memory cell state is updated by obtaining equations (13) and (14). Finally, the hidden state or the cell output is got by equation (15). It is clear that the LSTM is different from RNN because the $h_{t-1}$ is not entirely used in each time step *t* in the LSTM while the RNN is. In addition, the $c_{t}$ regulates the time dependency and information flow rather than the other way around. As a result, when these additive connections are combined with the forget gate $f_{t}$, the problems of vanishing and exploding gradients are alleviated. Taking together, the LSTM tackles the problem that appears in almost all the standard RNNs. Hence, in this study, LSTM is used to design a deep learning-based model for forecasting the time domain data of the Rabi antenna.

## 3 Results and discussion

### 3.1 For plasmonic circuit

Quantum communication involves transmitting information using the principles of quantum physics. This information is processed in the form of quantum bits, so-called qubits, which can be distributed within the network. There are many ways to transfer information within a network. One method of transmitting information is through the use of spin wave carriers. In spin waves, magnetic moments collectively move within a magnetically ordered material. It plays an important role in spintronics and is used as a spin current carrier that represents the angular momentum motion of the spin. Spin currents can be generated using surface plasmons. The movement of charges in surface plasmons generates electromagnetic fields both inside and outside the metal called surface plasmon polaritons. Surface plasmon polaritons form the basis of plasmonic circuits and can also be manipulated like photons, leading to the design of plasmonic circuits. Plasmonic circuits are effectively used to guide and confine light below the diffraction limit.

**Figure 3:**
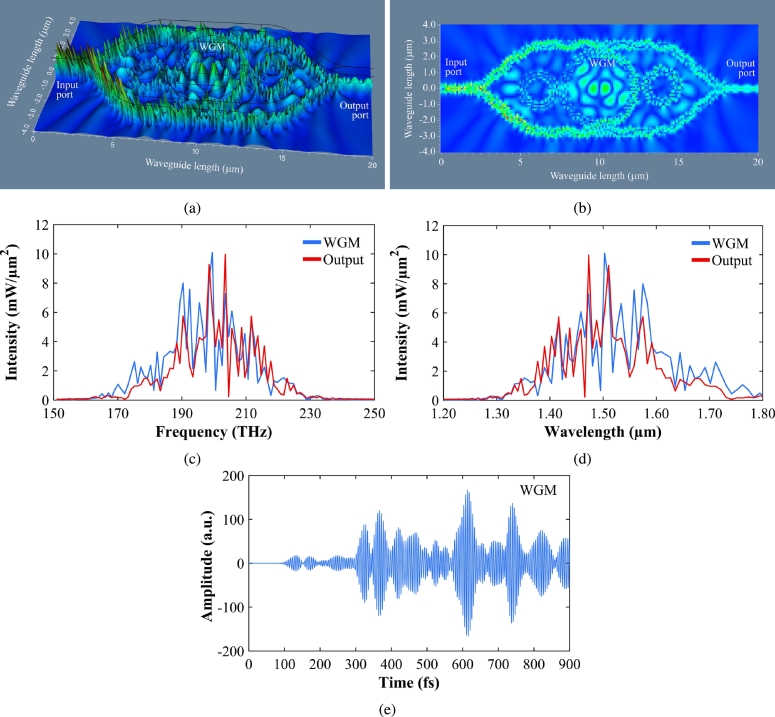
(a) Transmission profile. (b) Electron distribution. (c) WGM results with frequency domain graph of WGM and output port. (d) WGM results with wavelength domain graph of WGM and output port. (e) WGM results with time domain graph of WGM and output port.

**Figure 4:**
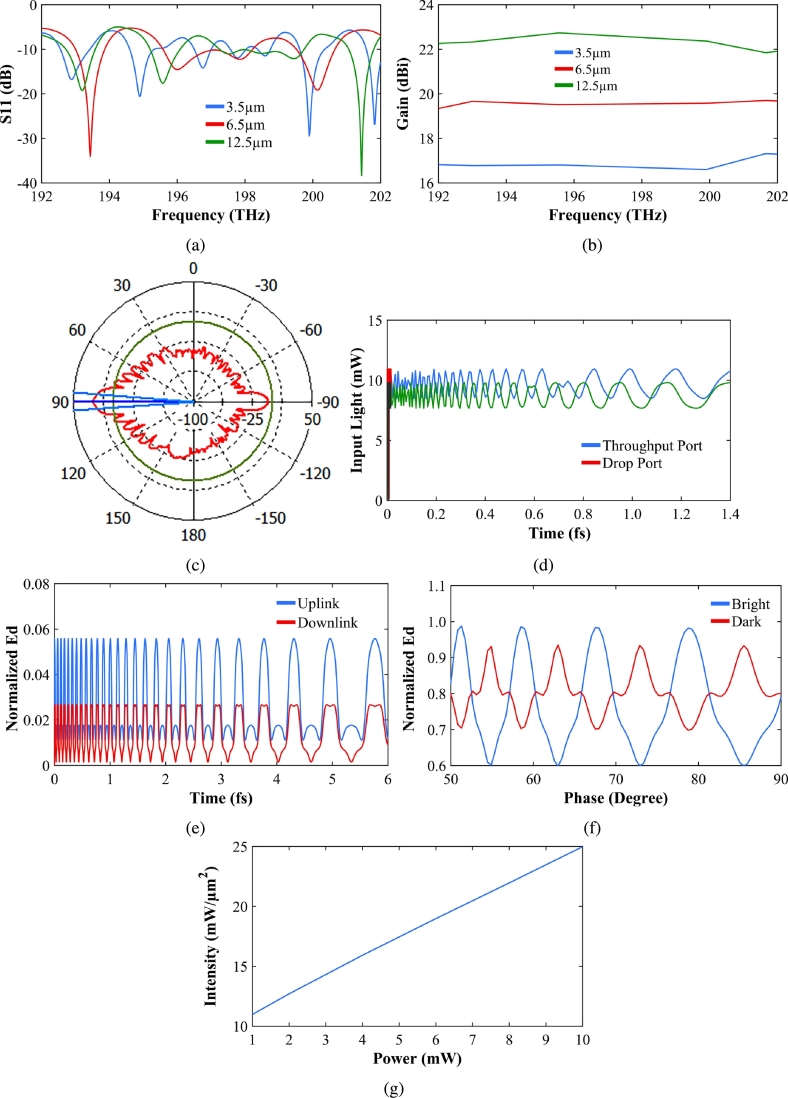
(a) Compared antenna S11 plots. (b) Compared antenna Gain plots [maximum gain is 22.4 dBi when substrate height is 12.5 μm]. (c) Directivity is 24.2 dBi, at 193.22 THz. At this frequency the main lobe direction is at 90^∘^. The angular width (3 dB) is 8.3 degree. The result is plotted in CST microwave studio. (d) Rabi oscillation results confirm the two-level system oscillation. (e) Plot of electron spins and input power for quantum sensors that can be used for human mind, thoughts, behavior and action/reaction sensors electron spins of $1.6\;{\mu \text{m}}^{-2}{\left(\text{mW}\right)}^{-1}$. (f) Plot of electron spins and input power for quantum sensors that can be used for human mind, thoughts, behavior and action/reaction sensors Entanglement of $1.6\;{\mu \text{m}}^{-2}{\left(\text{mW}\right)}^{-1}$. (g) Plot of electron spins and input power for quantum sensors that can be used for human mind, thoughts, behavior and action/reaction sensors sensitivity (bottom) of $1.6\;{\mu \text{m}}^{-2}{\left(\text{mW}\right)}^{-1}$.

The system is initially configured with optiFDTD. This system consists of panda rings embedded in the MZI. The input and throughput ports are on the top branch of the MZI and the add and drop ports are on the bottom branch. Structures are simulated to achieve WGM in the central ring. OptiFDTD results are then extracted and plotted using MATLAB. The MZI material is Silica/Silicon (Si). The center ring of microring is embedded with silver bars. The input is the polarized laser of wavelength 1.51 μm which is applied at the input port of the MZI. The input wave propagates half-half into the upper and branches of MZI. The light couples into the center ring of radius 2.26 μm and side rings of radius 1.0 μm. Again, light combines at the output port of the MZI. Fig. 1a shows the proposed system. The simulated results of the system are shown in Figs. 3a and 3b. Fig. 3a presents the electric field result and Fig. 3b is the intensity result. The WGM is formed due to the nonlinear effects of two side rings. The electrons are trapped with silver nano bars. This is given by the Drude model in equation (3). Figs. 3c, 3d, and 3e summarize the WGM and outport results in the frequency domain, wavelength domain, and time domain, respectively. In Fig. 3c the WGM frequency and MZI output port frequencies are 199.4 THz and 203.5 THz with intensity values of 10.08 mW/${\mu \text{m}}^{2}$, 9.97 mW/${\mu \text{m}}^{2}$ respectively. Fig. 3d represents the WGM wavelength and MZI output port wavelength of 1.50 μm and 1.47 μm respectively. The intensity values are slightly higher in magnitude at WGM. The same system is designed using CST (computer simulation technology) software. The substrate material is silicon of refractive index 11.0 [23]. The antenna is designed in a similar way to [23]. The optimized parameters are given in Table 1. The antenna is simulated for the frequency range of 192–202 THz. The waveguide port is assigned. The CST results are shown in Figs. 4a–4c. The antenna is simulated for different substrate heights as 3.5 μm, 6.5 μm and 12.5 μm. Table 2 shows the compared results for different substrate heights. Fig. 4a shows the reflection coefficient or return loss of the designed antenna. For good results, the S11 values below -10 are considered. The resonating frequencies are 193.22 THz, 195.58 THz, and 201.44 THz with a return loss of -20 dB, -18 dB, and -38 dB, respectively at 3 frequencies. The maximum achieved bandwidth is 0.86 THz. Fig. 4b shows the Gain of the designed Rabi antenna. From Figs. 4a, 4b, and Table 2, it is clear that the best results are achieved when substrate height is 12.5 μm. When substrate height increases antenna gain increases. The maximum achieved gain is 22.72 dBi and maximum directivity is 22.58 dBi and the efficiency is 64.8%. Fig. 4c shows the directivity plots of the designed antenna system at 193.22 THz resonating frequency. Two side lobes (east and west) in Fig. 4c indicate Rabi oscillation. Any two-level quantum system can be used to simulate qubits. This task is important in quantum computing. This is crucial as had it not been Rabi, it could not have been used for the human mind, thoughts, behavior, and action/reaction. Rabi vibration is the main process used to manipulate qubits. Rabi oscillation provides an important tool for implementing quantum gates that perform basic logic operations on two qubits. Figs. 4a–4c are plotted using CST software. The data is extracted from CST and then plotted in MATLAB to achieve better graphical results. The designed Rabi antenna is compared with existing literature antennas also as given in Table 3. Fig. 4d indicates the Rabi oscillation that confirms the two-level system. The system is simulated 10,000 times. The system collapses around 1.7 fs (femtosecond). The rabi oscillation is explained in more detail in [24]. Fig. 4e shows the electron spin for uplink and downlink communication. Where in this case the quantum signals are formed by the spin wave propagation, which is obtained at the through and drop ports, respectively. The output is the difference between the 90 phases, presented as the bright and dark signals in Fig. 4f. The output in terms of spin density sensitivity is leveled by spin up (0) and spin down (1). Each Rabi switching has 2-quantum bits(0,1), which randomly switch and localize on two sides of time, positive, and negative. Each piece of information has many quantum bits. However, the same information can be resonant and recovered promptly by the projection on two sides of time. The memory has never been filled up by the Hilbert space (time sequence) arrangement. Memory will be lost after death. Fig. 4g shows the sensor sensitivity which is calculated from the slope of intensity and input power. The obtained value is 1.66 ${\mu \text{m}}^{-2}$. The input power is varied from 1 mW to 10 mW. The gain, efficiency, and directivity plots for 12.5 μm substrate height are plotted. The proposed Rabi antenna has the highest gain in comparison to the existing antennas (Table 2). Efficiency is around 65% and also better than [25].

**Table 1:** Parameters used for Simulation [23], [24].

Parameter	Symbol	Value	Unit
Input wavelength	*λ*	1.51	μm
Input power	P	1–10	mW
Center ring radius	R1	2.26	μm
Small rings radius	R2	1.0	μm
Coupling coefficient	*κ*	0.60–0.70	
Si refractive index	*n*_*Si*_	3.47	
Silver refractive index	*n*_*Ag*_	0.14	
Silver bar length	L	0.75	μm
Silver bar width	W	1.5	μm
Silver bar thickness	D	0.55	μm
Metallic film area	A	1.55 × 3.0	${\mu \text{m}}^{2}$
Waveguide loss	*α*	0.5	dB.(mm)^−1^
Effective core area	*A*_*eff*_	0.30	${\mu \text{m}}^{2}$
Mass of electron	m	9.11 × 10^-31^	Kg
Electron charge	e	1.6 × 10^-19^	Coulomb
Permittivity of free space	*ϵ*_0_	8.85 × 10^-12^	Fm^−1^
Si dielectric constant	*ϵ*_*r*_	11.9

**Table 2:** Antenna result comparison.

Parameter	Substrate thickness		
	3.5 μm	6.5 μm	12.5 μm
Resonating frequency (THz)	193.39, 195.72, 197.95, 201.66	193.0, 195.3, 198.96, 201.44	193.22, 195.58, 201.44
Resonating wavelength (μm)	1.55, 1.53, 1.51, 1.48	1.55, 1.53, 1.50, 1.48	1.55, 1.53, 1.48
Bandwidth range (THz)	193.01-194.01=1.0 195.40-196.23=0.83 197.64-198.23=0.59 201.30-202.17=0.87	192.68-193.4=0.72 194.95-197.25=2.3 198.42-199.39=0.97 201.15-201.82=0.67	192.75-193.57=0.82 195.2-196.06=0.86 201.06-201.80=0.38
Gain (dB)	Max gain of 17.31, 16.78, 16.79, 16.60, 17.31	19.66, 19.44, 18.58, 20.08	Max gain 22.31 22.31, 22.72, 22.01
Directivity (dBi)	19.48	22.19	24.58
Efficiency radiation	Max. efficiency of 61.0%	Max efficiency of 64.8%	Max efficiency of 64.8%

**Table 3:** Comparison with existing antenna's.

Reference	Frequency	Gain	Directivity	Efficiency (%)
[26]	180–230 THz	10 dB (realized gain)	8 dBi	–
[27]	0.5–1.5 THz	12.67 dB	–	–
[28]	0.835, 0.635, 0.1 THz [Bw=24.3, 17.3 and 1.8 GHz]	16.37, 16.52 and 15.7	–	58.4, 52 and 52
[29]	190–200, BW=5 THz	–	8.6 dBi at 193.5 THz	–
[30]	180–200 THz, BW=20 THz	9.03, 9.08 [realized gain] 1550 nm	10.00, 10.32	92.25%, 92.89%, Area=1200*950 nm^2^
[31]	150–400 THz 193.5 (1550 nm), 229 (1310 nm), 315 THz (850 nm)	4.67, 7.26, 4.8	–	Foot print area=450*625 nm^2^
[32]	190–200, [190.9–198.1, BW=7.2 THz	–	9.57, 8.6, 7.85 dBi at 191, 193.5, 198 THz	–
[25]	170–230, 175, 185, 205, 220.	>9 dB	–	40%
[33]	180–220 THz 2.58% (192.5–197.3 THz)	–	8.34, 8.6, 9.69 dBi at 192.5, 193.5 and 197 THz	–
Proposed Rabi antenna	192–202 THz, max bandwidth of 0.86 THz	Max Gain 22.4 dB	Max directivity=24.6 dBi	64.8%

In this present work, the substrate thickness of 12.5 μm is selected. This selection reflects that the efficiency is maximum and values of Gain, bandwidth, directivity are more. When thickness increases, gain increases as well.

### 3.2 For deep learning circuit

The LSTM network was used to forecast the amplitude of time domain data from the Rabi antenna in this experiment. The data has 10,000 samples corresponding to 10,000 time-based steps. The data is then divided into training and testing subsets: train on the first 90% of the sequence and test on the remaining 10%. The training data is normalized to acquire zero mean and unit variance for a better prediction and to avoid the training from diverging. Standardize the testing data, it is also done at the prediction stage using the same parameters as the training data.

To anticipate the values of future time steps in a series, specify the replies to be training sequences with values shifted by a one-time step. At each time step of the input sequence, the LSTM network learns to expect the value of the next time step. Predictors are training sequences in which the last time step is not included. The next step is to build an LSTM regression network with 200 hidden units. The optimizer is Adam, with a number of epochs of 250, a learning rate of 0.001, and a learning rate schedule that decreases by a factor of 0.2 after 125 epochs. The gradient threshold is also set to 1, which prevents the gradients from growing. It should be noted that the hyperparameters and optimal structure of the proposed deep learning model are tuned using a grid-search-based method. The hyperparameters are reported based on the highest performance of the proposed deep learning prediction model.

**Figure 5:**
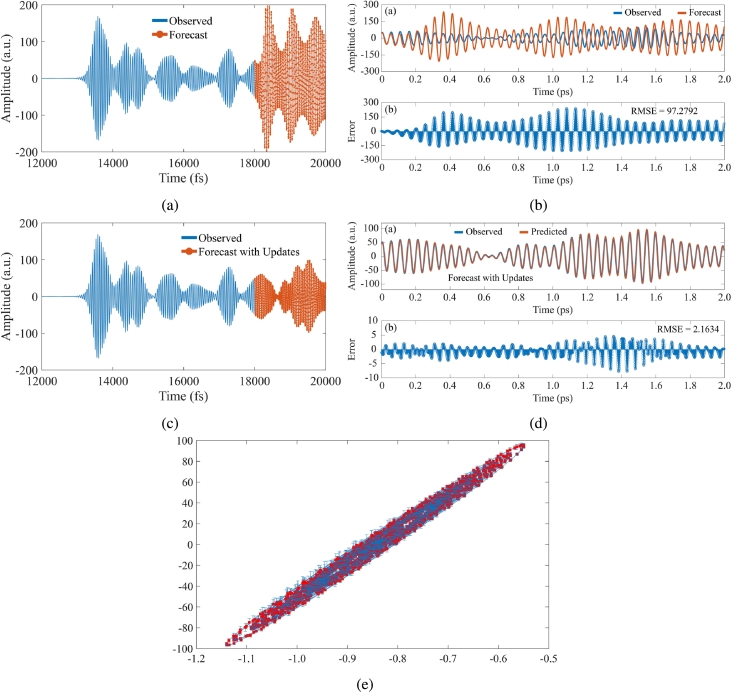
(a) Plot of training time series with the forecasted values. (b) Comparison of the forecasted values with the test data. (c) Plot of training time series with the forecasted values after updating the LSTM network. (d) Comparison of the forecasted values with the test data after updating the LSTM network. (e) Plot of error bars with *RMSE* = 2.3332(±0.2338).

After training the LSTM model, we used trained LSTM to predict the future time steps in a series using the test data as the input. Fig. 5a displays the training time series with the forecasted values. In Fig. 5a, the blue line is the observed or the historical data whereas the red line with the dot is the predicted future time steps in series. It is clear that the LSTM network can learn the characteristics of the time domain data from Rabi antenna to predict the next time steps using the historical data. Here, on the other hand, the LSTM network can also be used to explain the operation of the brain through the time domain data resulting from the Rabi antenna. Fig. 5b shows the comparison of the forecasted values with the test data with the root mean square error (RMSE) of 97.2792.

The network state is then updated with observed values, and the time series of time domain data is forecasted once more. The training time series with the anticipated values after updating the network is shown in Fig. 5c. The projected future time steps of time domain data diverge somewhat from the raw time domain data, as seen in Fig. 5c. In addition, after upgrading the LSTM network, we compare the anticipated values to the test data once more. With an RMSE of 2.1634, the result is shown in Fig. 5d. Finally, the plot of error bars with RMSE=2.3332(±0.2338) is depicted in Fig. 5e.

Furthermore, the results in Figs. 5a–5d demonstrate that the brain signal resulting in Rabi antenna probes can be learned and predicted using a deep neural network (i.e. LSTM). This indicates that the deep neural network and the bio-neural network are comparable in many ways, based on these discoveries. Besides, this study is the first time a deep learning circuit is designed to predict the brain signal based on the Rabi antenna probes and there is no previous publication related to this finding to the best of our knowledge. For knowledge-sharing and repeatability/reproducibility purposes, our implementation of the method is publicly available at https://github.com/nhattruongpham/Deep_Brain_SigNet.

## 4 Conclusions

In this article, we have utilized CST and optiFDTD software tools to design and simulate the Rabi plasmonic antenna system. The proposed plasmonic antenna has a silver rod in the central ring. This antenna has a maximum gain of 22.4 dBi and a maximum directivity of 24.6 dBi. The sensitivity of the sensor is 1.6. Rabi vibration is confirmed by a two-level system. Electron spins and quantum sensors show that they are very useful and applicable to the human mind, thoughts, behavior and action/reaction sensors. In other words, our current work is an extensive simulation Study and corresponding disease prediction model on human brain through the integration of deep neural network and biosensor Rabi antenna targeted at the human mind, thoughts, behavior and actions that might be helpful to detect neuro-degenerative/psychological disorders and security purposes (criminal detection).

While the use of LSTM for time series modeling is fundamental, there are many advanced deep learning methods available. However, in our framework, our main focus is on an integrative approach using multidisciplinary methods such as deep neural networks and biosensor Rabi antennas, applied to the biomedical domain based on simulation and prediction. This integrative approach is relatively new, and we are currently searching for stronger publicly available or newly generated related data to enhance our method and make it more realistic and efficient. Nonetheless, our proposed method can be scaled to design biosensor Rabi antenna devices that researchers can use in their experimental research for disease prediction and monitoring. Moreover, our proposed model can predict human minds, thoughts, behaviors, as well as actions and reactions, which can greatly aid in the diagnosis of various neurodegenerative and psychological diseases (such as Alzheimer's and dementia) and to some extent, for security purposes. Nevertheless, further experimental validation using real-life public data is necessary to extend this approach.

Moreover, in practical applications, noises are inevitable in sensor data and can affect the results of the deep neural network prediction model. As a result, the sensor network data denoising method [34] should be considered and investigated in future work. Future work has the potential to develop Rabi chips to create humanoid robots. Stereo sensors and networks need to be incorporated into systems that enable a realistic humanoid experience. Sequential filtering processes can also produce smarter humanoids.

## CRediT authorship contribution statement

Nhat Truong Pham: Performed the experiments; Analyzed and interpreted the data; Contributed reagents, materials, analysis tools or data; Conceived and designed the experiments; Wrote the paper.

Montree Bunruangses: Conceived and designed the experiments; Performed the experiments; Wrote the paper.

Phichai Youplao; Anita Garhwal: Performed the experiments; Analyzed and interpreted the data; Contributed reagents, materials, analysis tools or data.

Arup Roy: Contributed reagents, materials, analysis tools or data.

Sarawoot Boonkirdram; Muhammad Arif Jalil: Performed the experiments; Analyzed and interpreted the data.

Preecha Yupapin; Kanad Ray; Jalil Ali; Shamim Kaiser; Mufti Mahmud; Saurav Mallik; Zhongming Zhao: Conceived and designed the experiments; Wrote the paper.

## Funding statement

Z.Z. was partially supported by 10.13039/100004917Cancer Prevention and Research Institute of Texas (CPRIT RP180734) and the Precision Health Chair Professorship fund. The funders did not participate in the study design, data analysis, decision to publish, or preparation of the manuscript.

## Declaration of Competing Interest

The authors declare no conflict of interest.

## Data Availability

Data included in article/supp. material/referenced in article.
